# Soluble CCR2 gene therapy controls joint inflammation, cartilage damage, and the progression of osteoarthritis by targeting MCP-1 in a monosodium iodoacetate (MIA)-induced OA rat model

**DOI:** 10.1186/s12967-022-03515-3

**Published:** 2022-09-23

**Authors:** Hyun Sik Na, Seon-Yeong Lee, Dong Hwan Lee, Jin Seok Woo, Si-Young Choi, Keun-Hyung Cho, Seon Ae Kim, Eun Jeong Go, A Ram Lee, Jeong-Won Choi, Seok Jung Kim, Mi-La Cho

**Affiliations:** 1 Lab of Translational ImmunoMedicine, Catholic Research Institute of Medical Science, College of Medicine, The Catholic University of Korea, Seoul, 06591 Republic of Korea; 2 Department of Orthopedic Surgery, Uijeongbu St. Mary’s Hospital, College of Medicine, The Catholic University of Korea, 271, Cheonbo-Ro, Uijeongbu-si, Gyeonggi-do 11765 Republic of Korea; 3 Department of Biomedicine & Health Sciences, College of Medicine, The Catholic University of Korea, 222, Banpo-daero, Seocho-gu, Seoul, 06591 Republic of Korea; 4 Department of Medical Lifescience, College of Medicine, The Catholic University of Korea, Seoul, 222, Banpo-daero, Seocho-gu, Seoul, 06591 Republic of Korea

## Abstract

**Background:**

Osteoarthritis (OA) is the most common type of degenerative arthritis and affects the entire joint, causing pain, joint inflammation, and cartilage damage. Various risk factors are implicated in causing OA, and in recent years, a lot of research and interest have been directed toward chronic low-grade inflammation in OA. Monocyte chemoattractant protein-1 (MCP-1; also called CCL2) acts through C–C chemokine receptor type 2 (CCR2) in monocytes and is a chemotactic factor of monocytes that plays an important role in the initiation of inflammation. The targeting of CCL2–CCR2 is being studied as part of various topics including the treatment of OA.

**Methods:**

In this study, we evaluated the potential therapeutic effects the sCCR2 E3 gene may exert on OA. The effects of sCCR2 E3 were investigated in animal experiments consisting of intra-articular injection of sCCR2 E3 in a monosodium iodoacetate (MIA)-induced OA rat model. The effects after intra-articular injection of sCCR2 E3 (fusion protein encoding 20 amino acids of the E3 domain of the CCL2 receptor) in a monosodium iodoacetate-induced OA rat model were compared to those in rats treated with empty vector (mock treatment) and full-length sCCR2.

**Results:**

Pain improved with expression of the sCCR2 gene. Improved bone resorption upon sCCR2 E3 gene activation was confirmed via bone analyses using micro-computed tomography. Histologic analyses showed that the sCCR2 E3 gene exerted protective effects against cartilage damage and anti-inflammatory effects on joints and the intestine.

**Conclusions:**

These results show that sCCR2 E3 therapy is effective in reducing pain severity, inhibiting cartilage destruction, and suppressing intestinal damage and inflammation. Thus, sCCR2 E3 may be a potential therapy for OA.

## Background

Osteoarthritis (OA) is the most common joint disorder in modern society, especially as the number of elderly patients continues to increase; it affects the entire joint with cartilage destruction at its core [1]. As a multifactorial disease, there are various risk factors associated with OA, such as age, obesity, alignment, and mechanical loading, and in recent years, many studies and much interest have been directed toward chronic low-grade inflammation in OA [1, 2]. As interest in the treatment of early OA is gradually increasing, therapeutic drugs are being researched for use in disease-modifying treatments, and accordingly, many different approaches to OA treatment are being applied [3]. This includes research into the role of cytokines and chemokines in the pathological process of OA, as well as related therapeutic drugs [4].

Monocyte chemoattractant protein-1 (MCP-1/CCL2) is a member of the C–C chemokine family and acts as a chemotactic factor for monocytes [5]. It also plays an important role in the initiation of inflammation through C–C chemokine receptor type 2 (CCR2) in monocytes [6]. Research into which therapeutic effects are obtained by blocking inflammation and tissue damage through CCL2–CCR2 is being conducted for various diseases, such as rheumatoid arthritis (RA), cancer, atherosclerosis, myocardial infarction, and OA [7–10]. Inflammation is induced, and cartilage damage occurs through CCL2–CCR2, which plays an important role in OA progression [11, 12].

To block the pathway through CCL2–CCR2 in RA, a method for targeting and blocking CCL2 and CCR2 has been studied. Many clinical trials have been conducted on monoclonal antibodies and small-molecule drugs, but these have not exhibited efficacy in treating RA [13]. As effective results have not been obtained for other diseases including RA, research on methods based on targeting the CCL2–CCR2 pathway is ongoing [9]. CCL2–CCR2 plays an important role in the progression of OA compared to other chemokine receptors [4, 24]. Thus, studies on OA treatments that target the CCL2–CCR2 pathway have been increasing. Xu et al. [11] sampled chondrocytes from OA patients and performed MCP-1 stimulation; they found increased expression of MCP-1, CCR2, and MMP-13, as well as the induction of apoptosis of OA chondrocytes. Based on these studies, various drugs that block CCL2–CCR2 as therapeutic agents for OA as well as for RA are being developed and tested.

Izhak et al. [22] showed that a fusion protein comprising 20 amino acids of the third extracellular (E3) domain of the CCL2 receptor and a soluble CCR2 (sCCR2) receptor selectively binds to CCL2 and CCL16, and its binding affinity is effective compared to the N-terminal region or a combination of the E3 domain and N-terminal region.

In this study, we evaluated the potential therapeutic effects the sCCR2 E3 gene may exert on OA. The effects of sCCR2 E3 were investigated in animal experiments consisting of intra-articular injection of sCCR2 E3 in a monosodium iodoacetate (MIA)-induced OA rat model. The anti-nociceptive effects were analyzed, and the anti-inflammatory effects were assessed by sampling the knee joint and small intestine. Through histological and immunohistochemical (IHC) analyses, the degree of damage to the cartilage and small intestine was evaluated, and the levels of pro-inflammatory cytokines and catabolic factors were determined.

## Methods

### Animals

Seven-week-old male Wistar rats weighing 180–250 g each were purchased from Central Lab Animal Inc. (Seoul, South Korea). A maximum of three animals was housed per cage in a room with controlled temperature (20–26 °C) and light (12-h light/dark cycle) conditions. The rats had free access to a gamma ray-sterilized diet (TD 2018S; Harlan Laboratories, Indianapolis, IN, USA) and autoclaved reverse osmosis water. All animal research procedures were conducted in accordance with the Laboratory Animals Welfare Act, the Guide for the Care and Use of Laboratory Animals, and the Guidelines and Policies for Rodent Experiments provided by the Institutional Animal Care and Use Committee (IACUC) of the School of Medicine, the Catholic University of Korea. The IACUC and Department of Laboratory Animals of the Catholic University of Korea, Songeui Campus accredited the Korean Excellence Animal Laboratory Facility in accordance with guidelines of the Korean Food and Drug Administration in 2017, and full accreditation by AAALAC International was achieved in 2018.

### Plasmid vector construction

The rat sCCR2 E3 DNA and rat sCCR2 full DNA were amplification using PCR and the following forward and reverse primers were used: for rat sCCR2 E3, 5′-GTA CGA AGC TTG ACC ACC TTC CAG GAA TTC TTG GGA ATG AGT AAC TGT GTG GTT GAC ATG CAC TTA-3′ and 5′-GGT TCC TGC AGG GCC TGG TCT AAG TGC ATG TCA ACC ACA CAG TTA CTC ATT CCC-3′; for rat sCCR2 full, 5′-GGG GAA GCT TAT GGA AGA CAG TAA TAT GTT ACC TC-3′ and 5′-GGG ACT GCA GCA ACC CAA CTG AGA CTT CTT GCT C-3′. For stability and activity of E3, E3 was conjugated with Fc, and the rat sCCR2 E3-Fc was inserted into the HindIII and XboI sites of the pSecTag2A vector. The rat sCCR2 full were inserted into the HindIII and Pst1 sites of the pSecTag2A vector.

### Induction of OA and treatment using the sCCR2 gene

Animals were randomly assigned to treatment or control groups before the study began. After anesthetization with isoflurane, the rats were injected with 3 mg/50 μL of MIA (Sigma, St. Louis, MO, USA) using a 26.5G needle inserted through the patellar ligament into the intra-articular space of the right knee. The intact knee joint was intra articular (IA) injected with 100 μg/0.9% normal saline 200 μL/Rat of Mock control vector, sCCR2-E3 or sCCR2-Full vector by electroporation at 3, 6, 9 and 12 day. Pain and weight bearing score were analyzed at 0, 4, 7, 11 and 13 day.

### Assessment of pain behaviour

Nociception in MIA-treated rats randomized to the different experimental groups was tested using a dynamic plantar aesthesiometer (Ugo Basile, Gemonio, Italy). The device is an automated version of the von Frey hair assessment system and is used to assess mechanical sensitivity. The assessment was conducted by placing each rat on a metal mesh surface in an acrylic chamber in a temperature-controlled room (20–26 °C), where it rested for 10 min before the touch stimulator unit was positioned under the animal. An adjustable angled mirror was used to place the stimulating microfilament (0.5 mm diameter) below the plantar surface of the hind paw. When the instrument was activated, a fine plastic monofilament advanced at a constant speed and touched the paw in the proximal metatarsal region. The filament exerted a gradually increasing force on the plantar surface, starting below the threshold of detection and increasing until the stimulus became painful, as indicated by withdrawal of the rat’s paw. The force required to elicit a paw-withdrawal reflex was recorded automatically and measured in grams. A maximum force of 50 g and a ramp speed of 25 s were used for all aesthesiometer tests.

### Assessment of weight bearing

Weight balance in MIA-treated rats was analyzed using an incapacitance meter (IITC Life Sciences, Woodland Hills, CA, USA). The rats were each allowed to acclimate for 5 min in an acrylic holder. Then, two feet were fixed to the pad, and the weight balance was measured for 5 s. Three measurements were repeated in the same manner. The weights on the unguided and guided legs were determined. The percentage weight balance was obtained by comparing legs with and without OA.

### Histopathological analyses

Knee joint and ileum of small intestine samples were collected from each group at 14 day post-MIA induction. The tissues were fixed in 10% formalin solution, decalcified using Decalcifying Solution-Lite (Sigma), and embedded in paraffin. Sections of 4- to 5-μm thickness were cut using microtome (Leica RM2235; Leica Microsystems, Wetzlar, Germany), dewaxed using xylene, dehydrated through an alcohol gradient, and stained with hematoxylin and eosin (H&E) and safranin O. Cartilage damage was scored as described previously [14]. For intestine analysis, loss of epithelium, crypt damage, depletion of goblet cells, and infiltration of inflammatory cells were assessed as described previously [15, 16]

### Immunohistochemistry analyses

Paraffin-embedded sections were incubated at 4 °C with the following primary polyclonal antibodies: anti-rat-interleukin (IL) 1 beta (IL-1β) (Abcam, Cambridge, UK), anti-rat-IL-6 (Abcam), anti-rat-matrix metalloproteinase 13 (MMP-13) (Abcam), anti-rat-MCP-1 (Abcam), anti-rat-CCR2 (Novus Biologicals, Littleton, CO, USA) and anti-rat-IL-17 (Abcam). Specific first antibody was diluted by DAKO antibody diluent (Dako, Carpinteria, CA, USA) and incubated overnight. Then, the samples were incubated with the respective secondary biotinylated antibodies, followed by 30-min incubation with streptavidin-peroxidase complex. Reaction products were developed using 3, 3-diaminobenzidine chromogen (Dako). Numbers of positive cells were counted in high-power fields (magnification: 400×) with the aid of NIH ImageJ software and averaged for three randomly selected fields per tissue section.

### In vivo micro-computed tomography imaging and analyses

Micro-computed tomography (CT) imaging and analyses were performed using a bench-top cone-beam-type in vivo animal scanner (mCT 35; Scanco Medical, Brüttisellen, Switzerland). The animals were imaged at settings of 70 kVp and 141 μA using a 0.5-mm-thick aluminum filter. The pixel size was 8.0 μm and the rotation step was 0.4°. Cross-sectional images were reconstructed using a filtered back-projection algorithm (NRecon software; Bruker microCT, Kontich, Belgium). For each scan, a stack of 286 cross-sections was reconstructed at 2000 × 1335 pixels. The femur bone volume and surface were analyzed.

### Isolation and culture of MSC

Adipose tissues were collected from OA patients (IRB NO. UC14CNSI0150) and were digested with type I collagenase (Cat. LS004197; Worthington Biochemical Products, Lakewood, NJ, USA). The isolated cells expanded for 2–3 passages and used for experiments, as reported previously [17].

### Transfection of mock and sCCR2-E3

Transfection of Mock and sCCR2-E3 DNA vector were transfected in mesenchymal stem cell (MSC) from fat of osteoarthritis patient. 2 × 10^5^ MSC were cultured with serum free DMEM for 12 h. The cells were transfected with 100 μg/mL sCCR2-E3 DNA vector with X-tremeGENE™ HP DNA transfection reagent (ROCHE, NJ, USA). After transfection 6 h, the MSC was stimulated with 100 ng/mL LPS and 10 ng/mL MCP-1 for 12 h.

### Real-time PCR

Total RNA isolated from human chondrocytes using TRIzol reagent (Molecular Research Center, Cincinnati, OH, USA) was used to synthesize cDNA. The relative expression of specific mRNAs was quantified via real-time PCR using Sensil FAST SYBR (Bioline, Taunton, MA, USA), and the following sense and antisense primers were used: *SRY-box transcription factor 9 (SOX9),* 5′-ACT TGC ACA ACG CCG AG-3′ (sense) and 5′-CTG GTA CTT GTA ATC CGG GTG-3′ (antisense); *MMP-1*, 5′-CTG AAG GTG ATG AAG CAG CC-3′ (sense) and 5′-AGT CCA AGA GAA TGG CCG AG-3′ (antisense); *MMP-3*, 5′-CTC ACA GAC CTG ACT CGG TT-3′ (sense) and 5′-CAC GCC TGA AGG AAG AGA TG-3’ (antisense).

### Statistical analyses

The results are expressed as the mean ± standard error of mean (SEM), which were obtained from three separate experiments. Statistical significance was determined via the Mann–Whitney U-test or analysis of variance with Bonferroni’s post-hoc test using GraphPad Prism (version 5.01; GraphPad Software, San Diego, CA, USA). *P* < 0.05 was considered to indicate statistical significance.

## Results

### sCCR2 regulates the pain threshold in MIA-induced OA rats

**Fig. 1:**
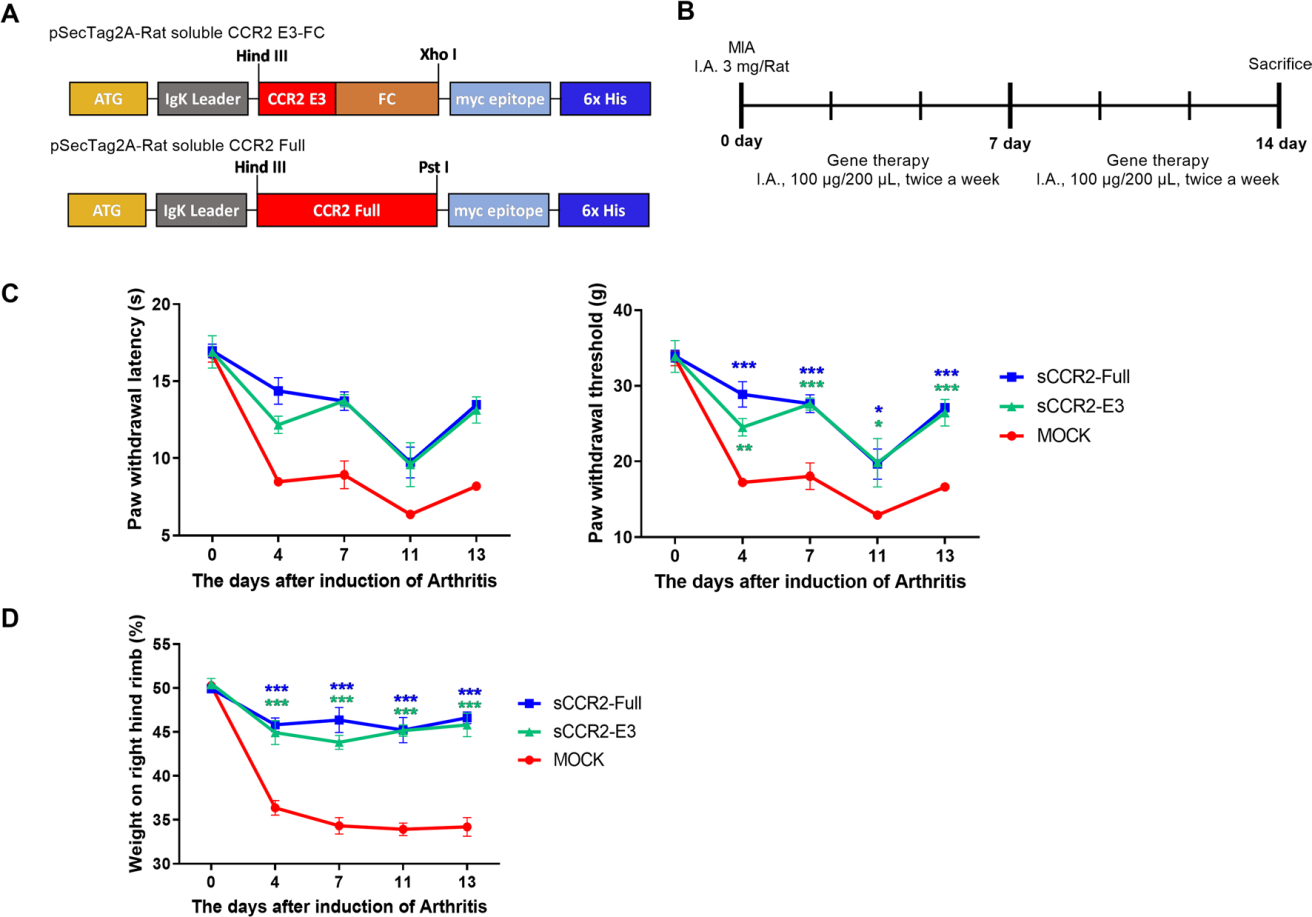
sCCR2 regulates the pain threshold in MIA rat. **A** A schematic diagram of soluble CCR2 E3 (sCCR2-E3) and soluble CCR2 full (sCCR2-Full) vector map. **B** A schematic diagram of the experimental protocols used demonstrating sCCR2-Full and sCCR2-E3 gene therapy in OA (N = 3). **C** Nociceptive testing was performed using a dynamic plantar esthesiometer, an automated version of the von Frey hair assessment procedure (PWL and PWT). **D** Weight bearing was performed using an in capacitance meter. Representative results from one of three independent experiments are shown regarding PWL, PWT and weight bearing for each group. The data are representative of at least three independent experiments. The data show the mean ± SEM of three independent experiments. (*p < 0.05, ** *p* < 0.01, *** *p* < 0.001)

To address the role of the sCCR2 gene in vivo (Fig. 1A), we injected copies of full-length sCCR2 (sCCR2-Full) or sCCR2-E3 into MIA-induced OA rats (Fig. 1B). Then, we measured paw withdrawal latency (PWL) and determined the paw withdrawal threshold (PWT) using a dynamic plantar aesthesiometer and compared outcomes between the OA and normal phenotypes. Both PWL and the PWT increased in both sCCR2 groups compared with the empty vector Mock group (Fig. 1C). Also, the weight bearing ability improved in both the full-length sCCR2 and sCCR2-E3 groups compared with the Mock group (Fig. 1D). These results indicate that both the full-length sCCR2 and sCCR2 E3 genes have similar effects in terms of controlling pain.

### sCCR2 reduces bone resorption in MIA-induced OA rats

**Fig. 2:**
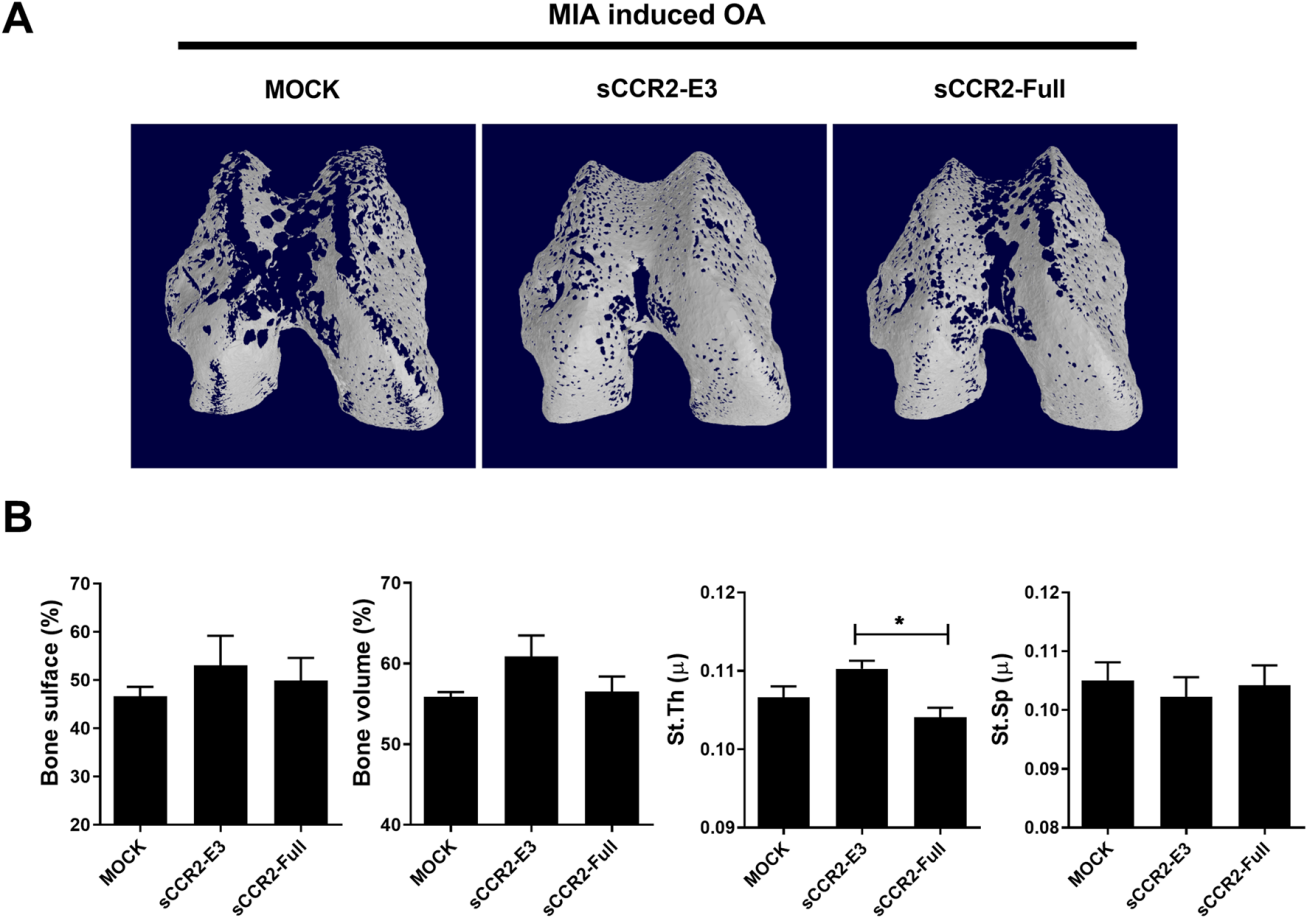
sCCR2 reduces bone resorption in MIA rat. **A** Bone samples were collected from MIA induced right knee at 14 day. Samples were scanned using mCT. **B** Bone volume, bone surface, structure thickness (st.th) and structure separation (st.sp) subchondral bone were measured to the BoneJ. The data show the mean ± SEM of three independent experiments. (*p < 0.05)

We investigated the role of the sCCR2 gene in bone resorption by performing bone analyses using micro-CT imaging of bone samples from Mock, sCCR2-E3, and sCCR2-Full (Fig. 2A). The bone surface, bone volume, and structure thickness of subchondral bone (St. Th) were increased in the sCCR2 E3 group, indicating less bone loss compared with the Mock and full-length sCCR2 groups (Fig. 2B).

### sCCR2 reduces cartilage damage in MIA-induced OA rats

**Fig. 3:**
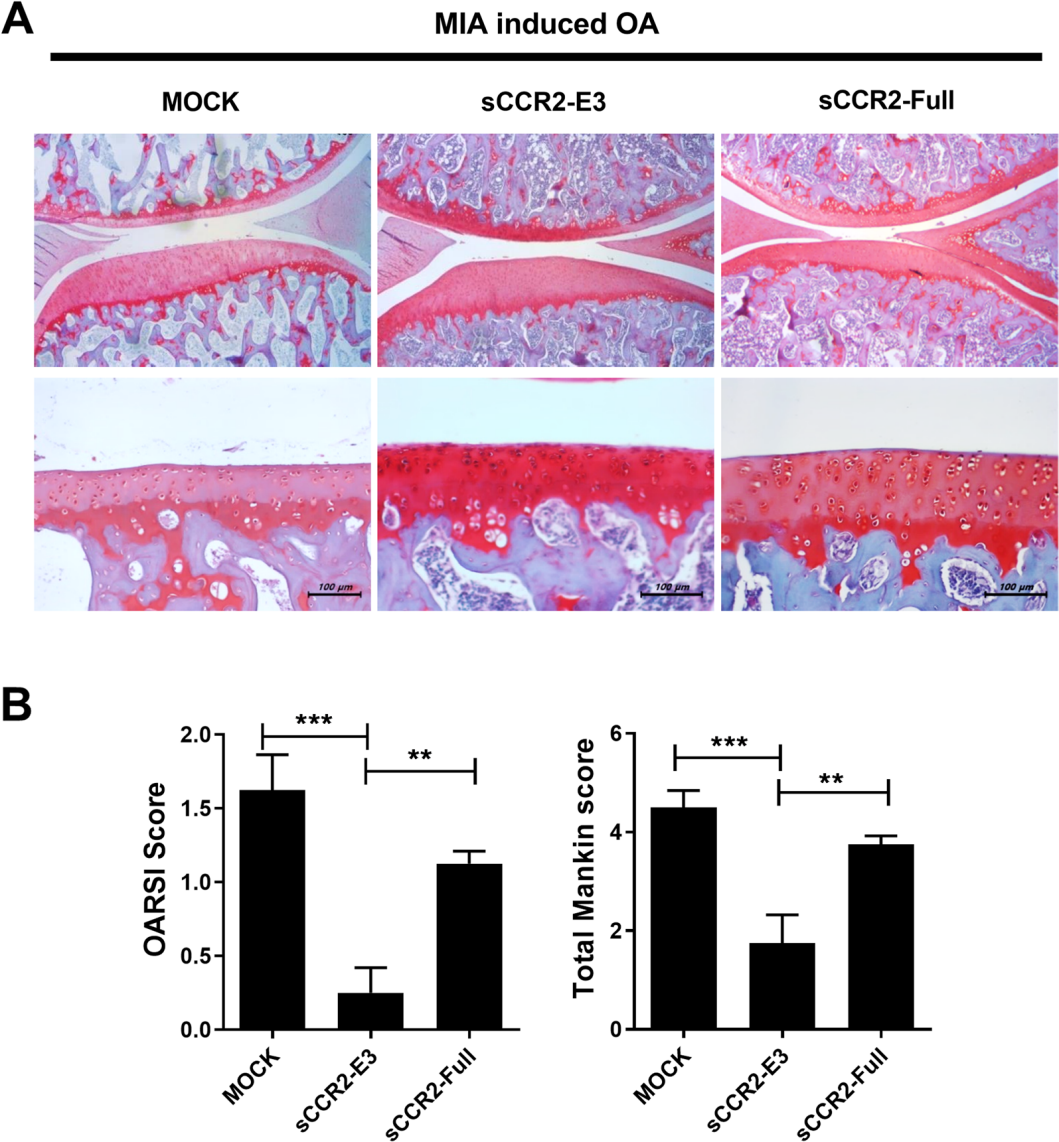
sCCR2 reduces depletion of proteoglycan in MIA rat. **A** Knee Joint tissue samples were collected from Mock and sCCR2-E3, sCCR2-Full rat at 14 day and subjected to stain with safranin O. **B** Stained tissues were assessed by Osteoarthritis Research Society International (OARSI) and Mankin scores. The data show the mean ± SEM of three independent experiments. (**p < 0.01, *** *p* < 0.001)

Knee joint tissue was collected from the Mock, sCCR2-E3, and sCCR2-Full groups, and safranin O staining was performed. Both the sCCR2-E3 and sCCR2-Full groups exhibited less proteoglycan depletion, with the sCCR2 E3 group exhibiting less proteoglycan depletion than the sCCR2-Full group (Fig. 3A). The results of safranin O staining were evaluated using the Osteoarthritis Research Society International (OARSI) score and Mankin score for each group. The scores were lower in both the sCCR2-E3 and sCCR2-Full groups than in the Mock group, and the sCCR2-E3 group had a lower score than sCCR2-Full group (Fig. 3B). These results suggest that sCCR2-E3 has a more protective effect against cartilage damage and bone destruction than full-length sCCR2.

### sCCR2 reduces inflammatory cytokines in the knee joint

**Fig. 4:**
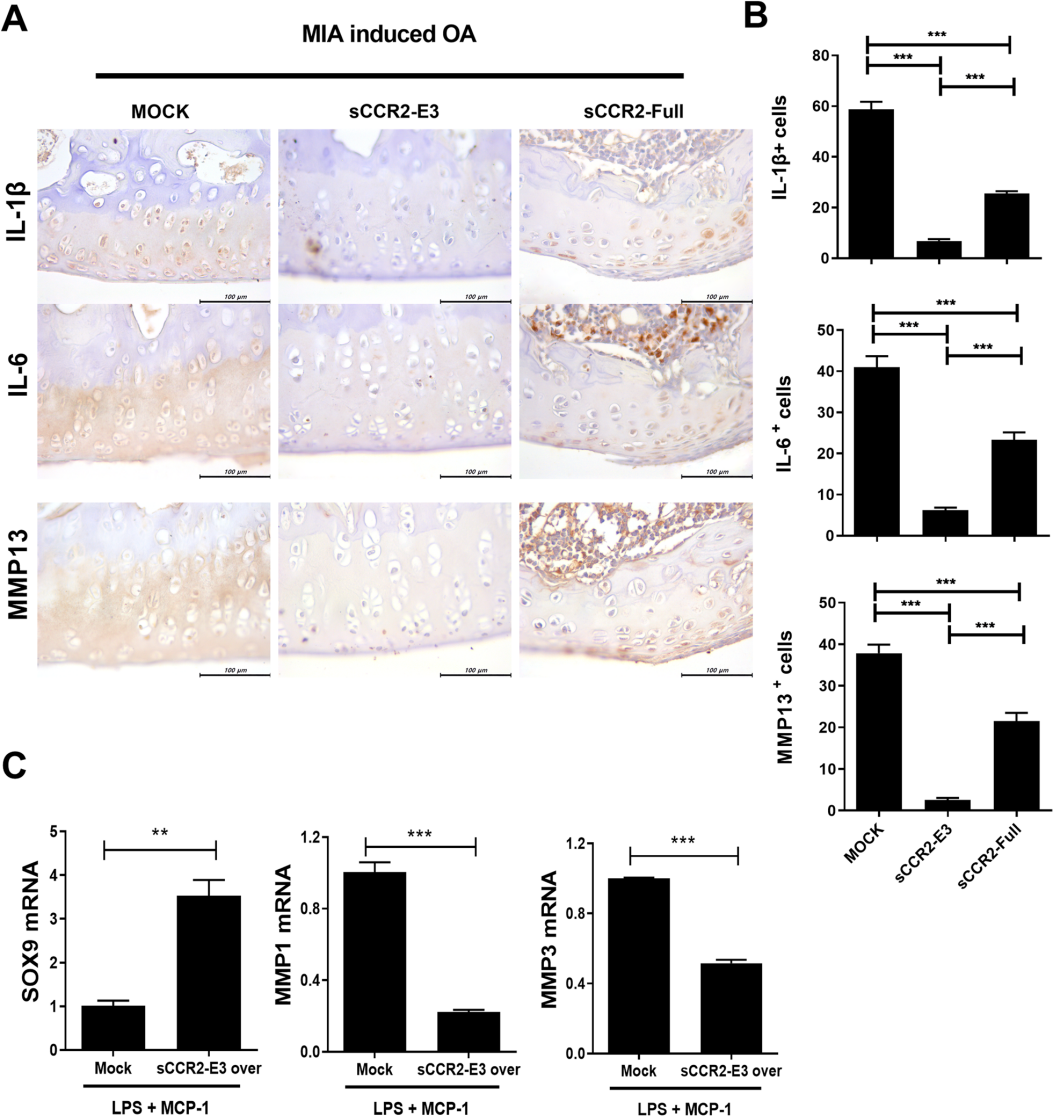
sCCR2 reduces inflammatory cytokines in knee joint. **A** Knee joint tissues from Mock and sCCR2-E3, sCCR2-Full rat were immunohistochemically stained with specific antibodies to IL-1β, IL-6 and MMP13. **B** Numbers of positive cells were counted in high-power fields (magnification: ×400) with the aid of NIH ImageJ software and averaged for three randomly selected fields per tissue section. Bars show the mean ± SEM results in three rats per group. **C** MSCs of OA patients were transfected Mock or sCCR2-E3. After 6 h, the media was changed and the cells stimulated with LPS 100 ng/mL and MCP-1 10 ng/mL for 12 h. The mRNA level of SOX9, MMP1 and MMP3 were determined using real-time. The data show the mean ± SEM of three independent experiments. (**p < 0.01, *** *p* < 0.001)

IHC staining was performed on the knee joint tissues collected from the Mock, sCCR2 E3 and sCCR2-Full groups. Inflammatory cytokines IL-1β and IL-6, and catabolic factor MMP-13 were analyzed from knee joint (Fig. 4A). There were less IL-1β-, IL-6-, and MMP-13-expression cells in the sCCR2-E3 than sCCR2-Full and Mock group (Fig. 4B). Next, we assessed chondrogenesis of Mesenchymal stem cells (MSCs) by sCCR2-E3. MSCs of OA patients were transfected Mock or sCCR2-E3 and then stimulated with lipopolysaccharide (LPS) and MCP-1. The mRNA level of SOX9, a chondrogenic factor, increased whereas those of MMP-1 and MMP-3, catabolic factors, decreased, in the sCCR2-E3 overexpressed MSCs (Fig. 4C). These data suggest that sCCR2-E3 induce MSC chondrogenesis by activating chondrogenic factors and inhibiting catabolic factors by scavenger of MCP1.

### sCCR2 reduces damage in the small intestine

**Fig. 5:**
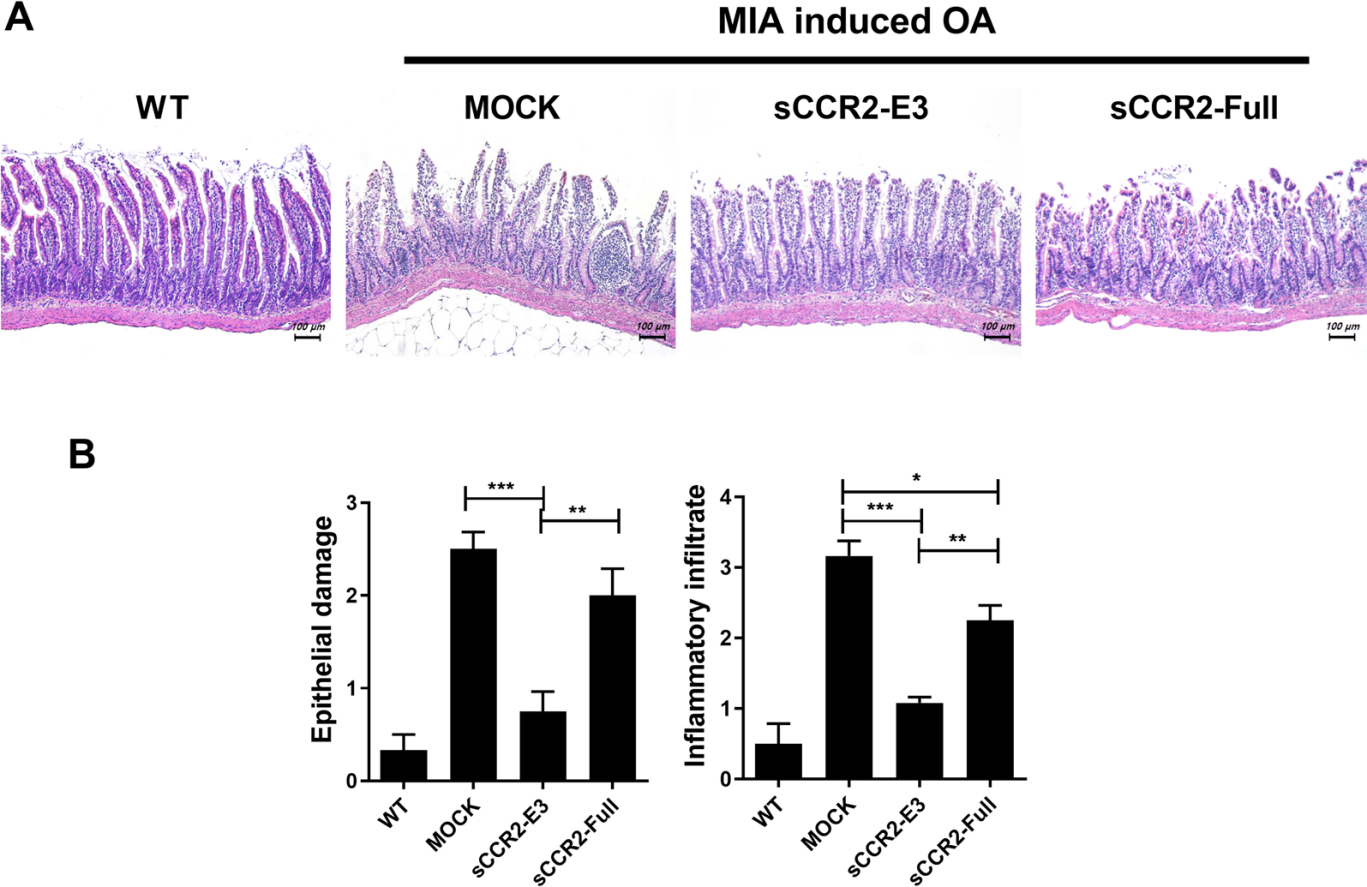
sCCR2 reduces damage in small intestine. **A** Small intestine tissues were acquired from Mock and sCCR2-E3, sCCR2 -Full rat at 14 day and subjected to stain with H&E. **B** Representative results from one of three independent experiments are shown regarding epithelial damage and inflammatory infiltrate score for each group. The data show the mean ± SEM of three independent experiments. (*p < 0.05, ** *p* < 0.01, *** *p* < 0.001)

**Fig. 6:**
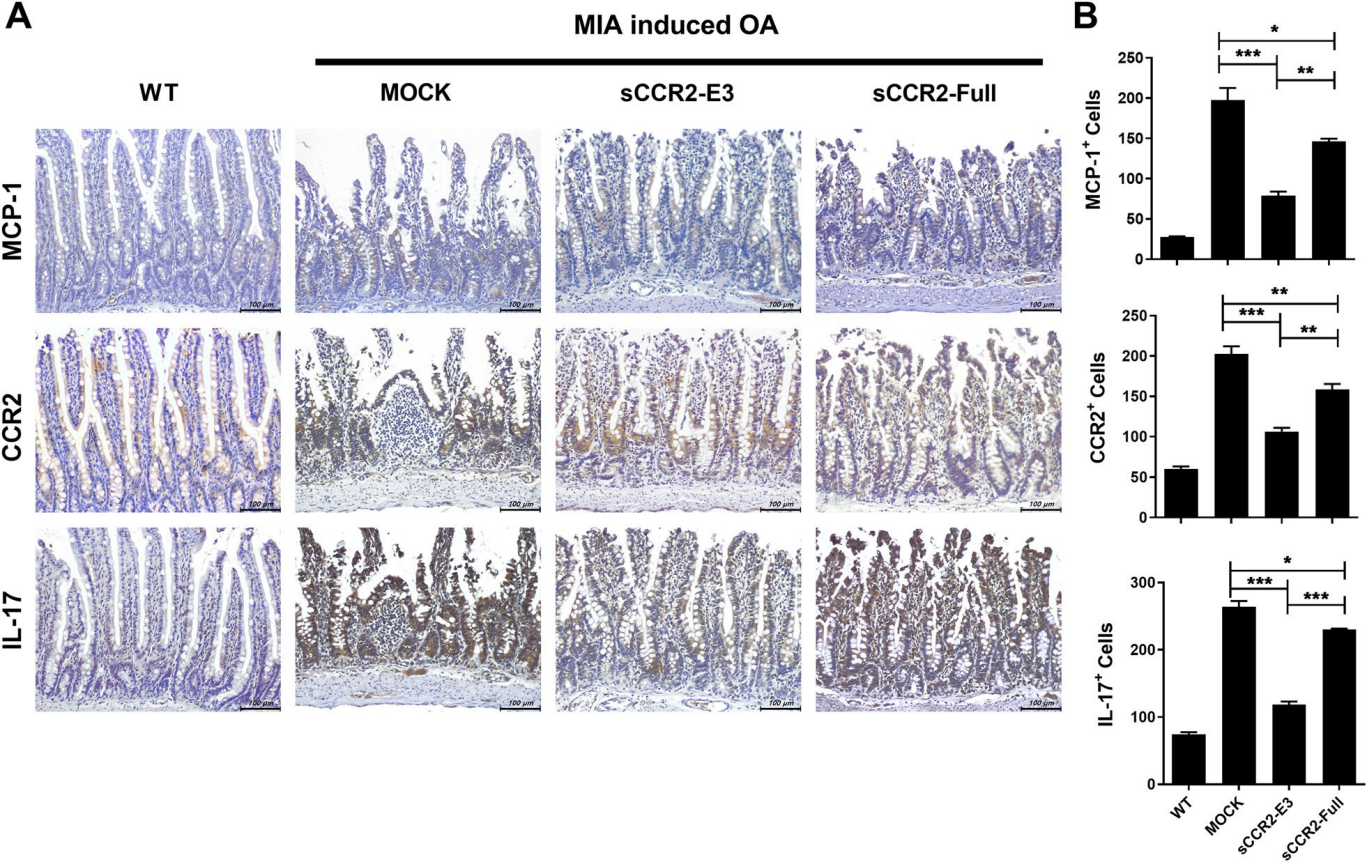
sCCR2 reduces inflammation in small intestine. **A** Small intestine tissues were immunohistochemically stained with specific antibodies to IL-17, MCP-1, CCR2. **B** Numbers of positive cells were counted in high-power fields (magnification: ×400) with the aid of NIH ImageJ software and averaged for three randomly selected fields per tissue section. Bars show the mean ± SEM results in three rats per group. The data show the mean ± SEM of three independent experiments. (*p < 0.05, ** *p* < 0.01, *** *p* < 0.001)

The inflammatory and nociceptive signals from a joint are transmitted to the brain through an afferent arc. The thus affected brain initiates a change in the acetylcholine/epinephrine balance through the efferent arc, which causes inflammation in the intestine [18, 19]. H&E staining showed that the sCCR2 gene exerted protective effects against intestine damage, and the sCCR2 E3 gene was more effective than sCCR2-Full (Fig. 5A). Intestinal damage was determined by evaluating epithelial damage and the infiltration of inflammatory cells. The sCCR2 E3 gene had better protective effects compared to the sCCR2-Full gene (Fig. 5B). To confirm the expression of inflammatory cytokines, we performed IHC staining of the small intestine villi (Fig. 6A). The number of MCP-1-, CCR2-, and IL-17-positive cells was markedly decreased in the sCCR2-E3 group (Fig. 6B). These results suggest that the sCCR2 E3 gene has protective effects against intestinal damage.

## Discussion

MCP-1/CCL2 is a chemokine that plays an important role in recruiting monocytes/macrophages and recruits monocytes to the site of inflammation through CCR2 [5]. Inflammatory responses through the CCL2–CCR2 pathway and associated damage cause various diseases such as auto-immune disorders including RA, cancer, atherosclerosis, myocardial infarction, viral infections, and OA.

There are two forms of CCR2, CCR2A and CCR2B, which have structural differences in the C-terminal tails; each form has a different mode of action and mechanism [20]. The expression of CCR2A was increased by CCL2 in an experiment on synoviocytes extracted from RA patients [21]. The binding affinity of CCR2 depends on the extracellular region, with the N-terminal region and E3 domain playing important roles [22–24].

CCR2 has dual functions. Blocking CCR2 leads to improvement in clinical signs and histological scores in the early phase of OA (days 0 to 15) but aggravates clinical and histological signs in the delayed phase (days 21 to 36), the latter which is caused by a humoral immune response involving regulatory T cells [25]. In addition, collagen-induced arthritis in CCR2-null mice exhibited a more severe RA pattern, and it was confirmed that CCR2 also plays a protective role in RA [26]. As such, CCR2 has both pro- and anti-inflammatory effects, and special care is required when developing therapeutic drugs that target this protein [8].

Recently, the roles of cytokines and chemokines in OA pathogenesis have garnered increasing attention. Unlike RA, CCL2–CCR2 plays an important role in the progression of OA compared to other chemokine receptors [4, 27]. Thus, studies on OA treatments that target the CCL2–CCR2 pathway have been increasing. Xu et al. [11] sampled chondrocytes from OA patients and performed MCP-1 stimulation; they found increased expression of MCP-1, CCR2, and MMP-13, as well as the induction of apoptosis of OA chondrocytes. Based on these studies, various drugs that block CCL2–CCR2 as therapeutic agents for OA as well as for RA are being developed and tested. As mentioned above, blockade using various molecules such as monoclonal antibodies or soluble receptors of CCL2 and CCR2 in RA has been effective in many animal experiments but has not obtained therapeutic effects in clinical trials [13]. There may be various explanations depending on each experiment, but the redundancy of action between chemokines and chemokine receptors composed of multiple ligands and receptors is likely the major cause [28]. Because of this redundancy, when a single ligand or receptor is targeted, the specificity decreases, leading to ineffective results [29, 30]. Izhak et al*.* [22] demonstrated through experiments that a fusion protein comprising as few as 20 amino acids of the E3 domain of the CCL2 receptor increases binding affinity compared to conventional CCR2 antagonists. In addition, in animal experiments, circulating blood levels of CCL2 increased only 1.8-fold after E3-Ig administration, indicating that E3-Ig can act as a more effective therapeutic agent. By contrast, a 2000-fold increase was observed in other studies using anti-CCL2 monoclonal antibodies [22, 31]. Based on these results, researchers have been able to obtain meaningful results by conducting experiments in which sCCR2 E3 gene therapy was applied to an OA model.

Transfection of the sCCR2 E3 gene into MSCs sampled from an OA patient was followed by treatment with LPS and MCP-1. It was confirmed that SOX9 increased whereas MMP-1 and MMP-3 decreased. This means that the sCCR2 E3 gene induced a chondrogenic effect and inhibited catabolic factors by blocking the action of MCP-1 in MSCs. Thus, MCP-1 is increased in OA and induces a pathologic condition, whereas CCR2 inhibits OA pathogenesis by blocking this action of MCP-1.

In OA patients, the suppression of inflammation and the control of pain play very important roles in the quality of life. Fundamentally, treatment with nonsteroidal anti-inflammatory drugs targets inflammation but has an insufficient effect on controlling joint cartilage destruction, so a treatment that simultaneously treats pain and cartilage destruction is urgently needed. In a previous OA surgical model, MCP-1 and CCR2 increased significantly in L3–L5 dorsal root ganglia, which govern pain signals from peripheral joints, and the calcium response in neurons increased significantly upon MCP-1 stimulation. Comparison of the CCR2-null and CCR2-antagonist groups with the wild-type (WT) destabilization of the medial meniscus (DMM) group confirmed that there was a significant decrease in pain and an increase in travel distance. This further confirms that the regulation of MCP-1/CCR2 can help with controlling pain in the peripheral joints [32]. In this study, based on the above results, we attempted to confirm the efficacy of controlling MCP-1 in pain reduction and cartilage protection. To this end, we conducted experiments comparing Mock, full-length sCCR2, and sCCR2 E3 gene therapy in an OA rat model, where MIA was administered to the knee joint to maximize pain and joint destruction. PWT, PWL, and weight-bearing measurements showed that the pain severity decreased, and there was no difference between the groups receiving full-length sCCR2 and sCCR2 E3 gene therapy. Through micro-CT analyses of the three groups, it was confirmed that bone resorption was decreased in the sCCR2 E3 group, with a greater decrease observed in the full-length sCCR2 group. When the knee joint tissues of the three groups were sampled and stained with safranin O, proteoglycan depletion was less severe in the sCCR2 E3 group compared to the full-length sCCR2 group. The OARSI and Mankin scores were also lower, confirming that cartilage destruction was prevented more effectively. In analyses of the inflammatory cytokines and catabolic factors IL-1β, IL-6, and MMP-13 based on IHC staining of samples from the three groups, the sCCR2 E3 group exhibited a greater decrease in levels compared to the full-length sCCR2 group, indicating that sCCR2 E3 had a more effective anti-inflammatory effect. Other previous studies have demonstrated that CCL2–CCR2 is associated with OA and plays a key role in the inflammatory response; however, no clear evidence on its association with chondropathy has been provided. MiotlaZarebska et al. performed histological analyses and scoring of CCL2^−/−^ and CCR2^−/−^ mice after DMM, but no significant differences with the WT mice were observed [12]. Xu et al. [11] injected a CCR2 antagonist (Sigma) in an MIA-induced OA rat model, but there was no significant improvement based on micro-CT analyses and the pathology score. In this study, we demonstrated through improvement in micro-CT outcomes and OARSI and Mankin scores that sCCR2 E3 gene therapy can prevent cartilage destruction.

In each of the four groups (WT, Mock, sCCR2, full-length sCCR2), the small intestine was sampled at 14 days after treatment administration, and the degree of damage according to the levels of epithelial damage and inflammatory cell infiltration was measured. The least damage was observed in the sCCR2 E3 group. Also, the expression of MCP-1, CCR2, and IL-17 in the intestine was confirmed through IHC staining, with the sCCR2 E3 group exhibiting the greatest inhibition. Through this, we demonstrated that intestinal damage and inflammation were most suppressed via sCCR2 E3 gene therapy targeting the knee joint. Regarding the connection between the knee joint and intestine, based on existing studies, the experiment was conducted based on expectations of a connection via the joint-brain-intestine pathway. When joint inflammatory and nociceptive signals are produced, they are transmitted to the brain through an afferent arc, and the brain stimulates the vagus nerve through an efferent arc. Then, acetylcholine is secreted, resulting in a cholinergic anti-inflammatory response [33–35]. In this process, the afferent arc is stimulated by cytokines secreted from the joint, with CCL2 expected to play a key role. Although the role of CCL2 has not yet been demonstrated, the above speculation can be made based on reports of disruption in the integrity of the blood–brain barrier via the CCL2–CCR2 pathway [18, 36]. When the brain is stimulated by a joint pathology, it is predicted that changes in the intestinal immune environment will occur through changes in hormone homeostasis resulting from alterations in the acetylcholine/epinephrine balance [19, 36]. In this study, we were able to partially prove the association.

OA has a different immunopathology compared to RA as a low-grade inflammatory disorder, and the CCL2–CCR2 pathway plays a more important role in the former [27]. Therefore, CCL2–CCR2 blockage through sCCR2 E3 is expected to have better therapeutic effects in OA than in RA. In this study, we proved that sCCR2 E3 can block the CCL2–CCR2 pathway in OA because of its high binding affinity, which solves some of the redundancy problems between multiple ligands and receptors. However, because the inflammatory response within the knee joint is a function of many different multiple pathways, its effect may be limited in actual clinical trials [37]. The redundancy problem will need to be resolved to obtain a meaningful therapeutic effect, and various approaches and studies, such as multiple targeting, as well as a method for increasing the specificity between chemokines and ligands are required.

## Data Availability

All datasets generated for this study are included in the article.

## Abbreviations

OA: Osteoarthritis
MCP-1: Monocyte chemoattractant protein-1
CCR2: C–C chemokine receptor type 2
MIA: Monosodium iodoacetate
IHC: Immunohistochemical
IL: Interleukin
MMP-13: Matrix metalloproteinase 13
CT: Micro-computed tomography
SOX9: SRY-box transcription factor 9
PWL: Paw withdrawal latency
PWT: Paw withdrawal threshold
OARSI: Osteoarthritis Research Society International
LPS: Lipopolysaccharide
DMM: Destabilization of the medial meniscus
